# Pediatric Leukemia From an Orthopedic Perspective: A Case of Acute Lymphoblastic Leukemia Initially Managed as Septic Hip With Osteomyelitis

**DOI:** 10.7759/cureus.24103

**Published:** 2022-04-13

**Authors:** Antigoni Gkoudina, Christos Gekas, Marianna Polydorides, Georgios Graikos, Evgenia Papakonstantinou, Panagiotis Saloupis

**Affiliations:** 1 Orthopedics and Traumatology, General Hospital of Pella, Edessa, GRC; 2 Orthopedics and Traumatology, Ippokrateio General Hospital of Thessaloniki, Thessaloniki, GRC; 3 Pediatric Surgery, Ippokrateio General Hospital of Thessaloniki, Thessaloniki, GRC; 4 Orthopedics and Traumatology, General Hospital of Thessaloniki "Georgios Papanikolaou", Thessaloniki, GRC; 5 Pediatric Oncology, Ippokrateio General Hospital of Thessaloniki, Thessaloniki, GRC

**Keywords:** musculoskeletal manifestation, pediatric malignancy, septic arthritis, joint pain, acute lymphoblastic leukemia (all)

## Abstract

Acute lymphoblastic leukemia (ALL) represents the most common pediatric cancer accounting for about one-third of all malignancies in childhood. The differential diagnosis for a pediatric patient manifesting with joint pain and refusal to bear weight is wide and includes trauma, transient synovitis, septic arthritis, rheumatologic disorders, and malignancy. Overt complaints from the musculoskeletal system as the initial manifestation of ALL may present in up to 30% of cases with normal laboratory tests and without hepatosplenomegaly or lymphadenopathy, perplexing the establishment of a definite diagnosis. Herein, we report the case of a three-year-old male who presented with recurrent hip pain and fever masquerading as septic arthritis recalcitrant to intravenous (IV) antibiotics, irrigation, and debridement of the hip joint with a final diagnosis of acute lymphoblastic leukemia confirmed by bone marrow biopsy.

## Introduction

Acute lymphoblastic leukemia (ALL) displays the commonest pediatric malignancy, with a peak incidence among children aged one to four years [1]. According to literature, the incidence of children with ALL who initially present with dominant symptoms from the musculoskeletal system can range from 21% to 59%, and these patients are usually referred for orthopedic or rheumatologic consultancy [2]. Muscle pain, bone pain, joint pain, low back pain, limping, refusal to bear weight, and pseudoparalysis constitute the orthopedic manifestations of ALL that can mimic other orthopedic conditions, especially in cases of normal laboratory testing, contributing to a delay in clinical diagnosis and adequate treatment. Hereinafter, we present the case of a child with ALL that manifested with hip pain, limp, fever, normal radiographs, and non-characteristic initial laboratory data misidentified and managed as septic arthritis.

## Case presentation

**Figure 1:**
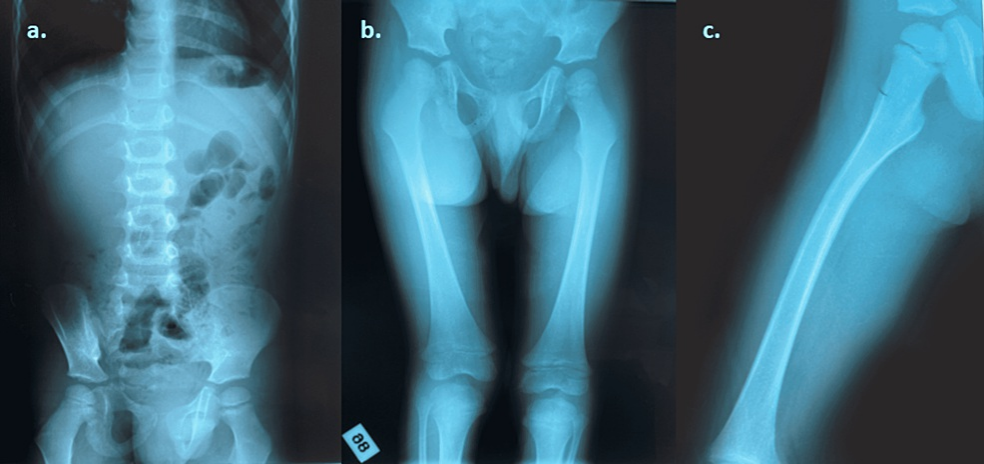
a) Plain abdominal X-ray with normal bowel gas pattern. b) Anteroposterior and c) frog-leg lateral hip X-ray without abnormal findings.

A three-year-old male presented to the pediatric emergency department with fever, acute abdominal pain, right hip pain, and refusal to bear weight for the past 10 hours. The parents mentioned that the child was discharged the previous day from a private pediatric center after being hospitalized for 12 days due to a presumed diagnosis of right upper femur osteomyelitis. Medical history was negative for migratory joint pain, morning stiffness, recent infection, easy bleeding, epistaxis, night sweats, fatigue, anorexia, and unintentional weight loss. The patient was extremely irritable, and his fever did not respond to the oral administration of paracetamol and ibuprofen at home. A thorough clinical examination at the emergency department revealed no skin rash, bruising, masses, or enlarged lymph nodes. Lung and heart auscultation were otherwise normal, except for sinus tachycardia (139 bpm). Abdominal examination revealed right lower quadrant (RLQ) abdominal tenderness in deep palpation without guarding, with normal bowel sounds. No distension, hepatomegaly, or splenomegaly was noted. During the musculoskeletal examination of the lower extremities, passive flexion and internal and external rotation of the right hip were significantly painful without visible erythema or swelling; however, passive range of motion was preserved. Neurologic examination was unremarkable with intact cranial nerves and normal muscle tone, strength, and sensation. Emergent laboratory investigation of complete blood count (CBC) highlighted a white blood cell (WBC) count of 14.7 × 10^3^/μL, low hemoglobin level, and a platelet (PLT) count of 472 × 10^3^/μL. The C-reactive protein (CRP) value was 5.8 mg/L (normal: <5 mg/L). Biochemical laboratory tests were within normal values, except for a significant lactate dehydrogenase (LDH) elevation (Table 1). Plain abdominal X-rays and anteroposterior and frog-leg lateral views of both hip joints were normal (Figure 1).

**Table 1:** Laboratory findings during hospitalization in a private pediatric center and hospital. RBC: red blood cells, Hct/HgB: hematocrit/hemoglobin, WBC: white blood cells, PLT: platelets, CRP: C-reactive protein, ESR: erythrocyte sedimentation rate, LDH: lactate dehydrogenase, post-op: postoperative

Private pediatric center							
	RBC	Hct/HgB	WBC (neutrophils (%)/lymphocytes (%))	PLT	CRP	ESR	LDH
Unit of measurement	× 10^6^/μL	Hct: %, Hb: g/dL	× 10^3^/μL	× 10^3^/μL	mg/dL	mm/hour	U/L
Reference values	4-5.3	Hct: 31.8-42.5, HgB: 10.5-14.5	5.4-15.8 (20-50/30-60)	150-450	<0.5	0-15	120-300
Admission day (day 1)	4.24	35.2/11.9	10 (55.5/41.7)	140	20.21	59	342
Day 2	3.95	32.9/11.3	8.1 (47.6/47.2)	100	10.2		
Day 3	3.83	31.8/11.1	10.5 (40/50)	120	9.6	98	
Day 5	3.55	29.5/10.3	10.7 (39/53)	145	6.1	95	394
Day 7	3.61	30.3/10.5	10 (35/59)	237	4.28	94	
Day 9	3.6	29.5/10.3	9.8 (33/60)	368	2.16		
Discharge day	3.6	29.8/10.2	13.4 (32/60)	409	1.35	15	469
Hospital							
Unit of measurement	× 10^6^/μL	Hct: %, Hb: g/dL	× 10^3^/μL	× 10^3^/μL	mg/L	mm/hour	U/L
Reference values	3.99-5.49	Hct: 31.8-42.5, HgB: 10.5-14.5	5.4-15.8 (12-60/31-78)	216-632	<5	0-10	<248
Emergency department	3.47	29/9.7	14.7 (36.9/58.6)	472	5.8		764
Post-op day 1	4.60	40.3/13.4	7.2 (46.3/51.2)	266	156.40		631
Post-op day 3	4.10	34.8/11.9	7.1 (31.7/64.1)	258	123.50		430
Post-op day 5	5.32	46.6/15.8	5.9 (20.3/70.3)	183	80.10		361
Post-op day 7	4.29	37.9/12.9	8 (17.1/79.2)	332	33.50	55	371

**Figure 2:**
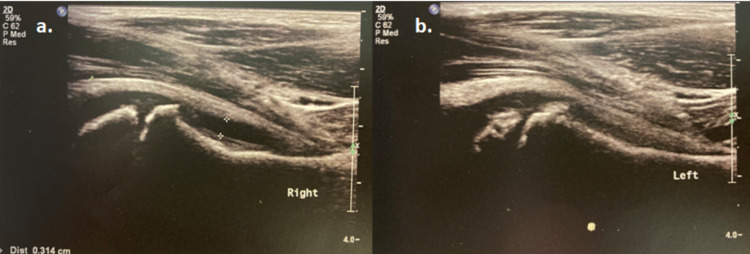
a) Right hip ultrasound demonstrating joint effusion (3.14 mm) (white marks). b) Normal left hip ultrasound.

**Figure 3:**
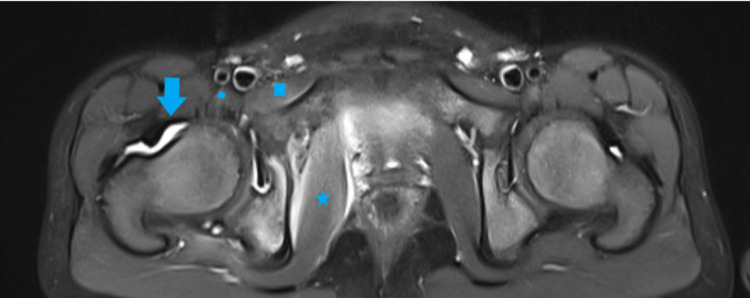
MRI axial T2 view with fat suppression depicting right hip joint effusion (arrow), synovial enhancement, edema, and enhancement of the obturator internus (star), pectineus (square), and psoas (dot) muscles. MRI: magnetic resonance imaging

A review of the patient’s previous hospitalization at the private pediatric center revealed that the child had presented with constant right hip pain that disturbed sleep and a limp. At the time of admission, the hematological profile was normal, but inflammatory markers were found to be elevated (Table 1). A subsequent hip ultrasound (u/s) confirmed the presence of right hip synovial thickening and joint effusion (Figure 2). Further workup for rheumatologic disorders was unremarkable (Table 2). Widal and Wright reaction proved to be negative as well. Additional investigation with magnetic resonance imaging (MRI) scan of the pelvis and hips demonstrated the presence of abnormal MR signal of the ilium, ischium, and pubis, accompanied by edema and increased enhancement of the obturator internus and externus, pectineus, iliacus, and psoas muscles with minimal intraperitoneal fluid. Right hip joint synovial enhancement, perisynovial edema, and joint effusion were also depicted with preserved joint space and normal femoral head MR signal (Figure 3). Osteomyelitis was strongly considered, and intravenous (IV) vancomycin and ceftriaxone were promptly and empirically administered and supplemented to the patient’s medication after collecting a blood culture specimen. After 12 days of hospitalization, the patient was eventually free of pain and able to ambulate, and inflammatory markers improved over time to nearly normal ranges (CRP: 1.35 mg/dL, ESR: 15 mm/hour). Blood cultures did not identify any bloodstream pathogen, and the patient was discharged from the private pediatric center with oral antibiotics.

**Table 2:** Advanced diagnostic testing. ASO: antistreptolysin O titer test, RF: rheumatoid factor, IgG: immunoglobulin G, IgA: immunoglobulin A, IgM: immunoglobulin M, Anti-dsDNA antibody: anti-double-stranded DNA, ANA: antinuclear antibody, CLIA: chemiluminescent immunoassay, IFA: indirect immunofluorescence assay

	ASO	RF	IgG	IgA	IgM	Anti-dsDNA	ANA	Total complement blood level	C3 level	C4 level	Widal reaction	Wright reaction
Unit of measurement	U/mL	IU/mL	mg/dL	mg/dL	mg/dL			U/mL	mg%	mg%		
Reference values	0-200	0.01-14	504-1464	27-195	24-210	Negative: <1	Negative: <1/80, weakly positive: 1/80, positive: >1/80	75-160	80-150	13-37		
Methodology						CLIA	IFA					
Result	47	8.5	585	77	68	Negative	Negative	158	145	35	Negative	Negative

**Figure 4:**
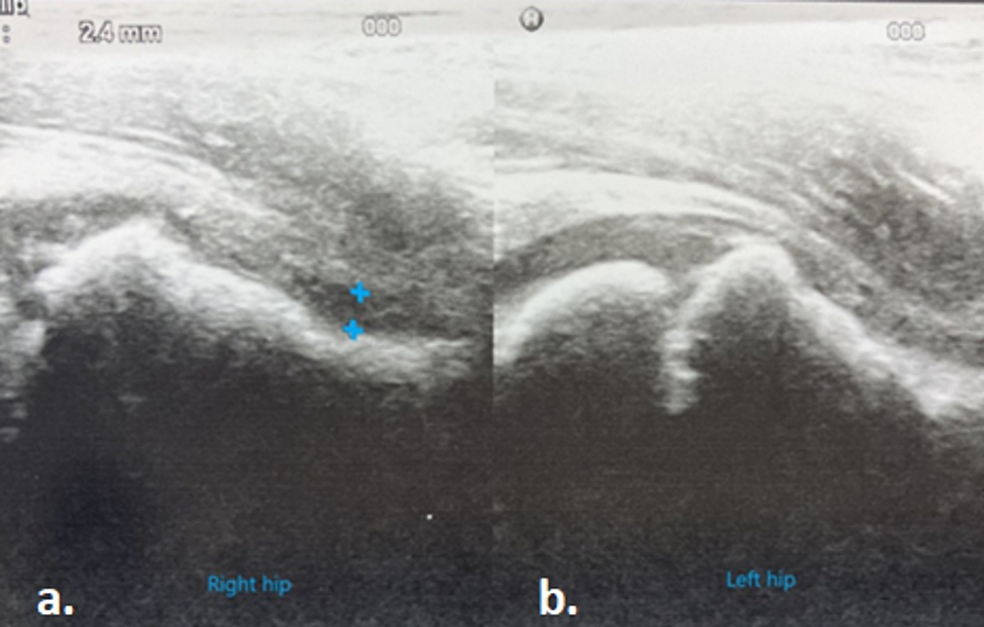
a) Right hip ultrasound indicating the presence of joint effusion (2.4 mm) (blue marks). b) Normal left hip ultrasound.

After the initial assessment at the emergency department of the hospital, the patient was referred to the pediatric surgery department for further evaluation of the abdominal pain. A blood specimen for culture was collected, and the patient was switched from oral to IV antibiotics with a single dose of ceftriaxone, piperacillin, and amikacin, along with a standard dose of vancomycin. Urgent abdominal u/s displayed a small volume of free intraperitoneal fluid, while right hip u/s demonstrated milder joint effusion (2.4 mm from 3.14 mm) compared to the initial right hip u/s from the private pediatric center (Figure 4). A subsequent investigation with computed tomography (CT) scan showcased traces of fluid in the pelvis minor with a few lymph nodes with a maximum diameter of 6 mm along the right iliac vessels. CT scan also detected muscle enlargement of the pectineus and obturator internus and externus muscle and a hypodense lesion into the mass of the latter on the right side. Βilateral inguinal lymph nodes with a maximum diameter of 7 mm were depicted, along with lytic lesions of the ilium, ischium, and pubis. Post-admission day, the child remained febrile (maximum axillary temperature: 38.8°C), and hip pain was resistant to analgesics; nonetheless, the RLQ pain had receded completely. An orthopedic surgery evaluation was requested, and a presumed diagnosis of septic arthritis was made according to the patient’s clinical examination and laboratory tests.

**Figure 5:**
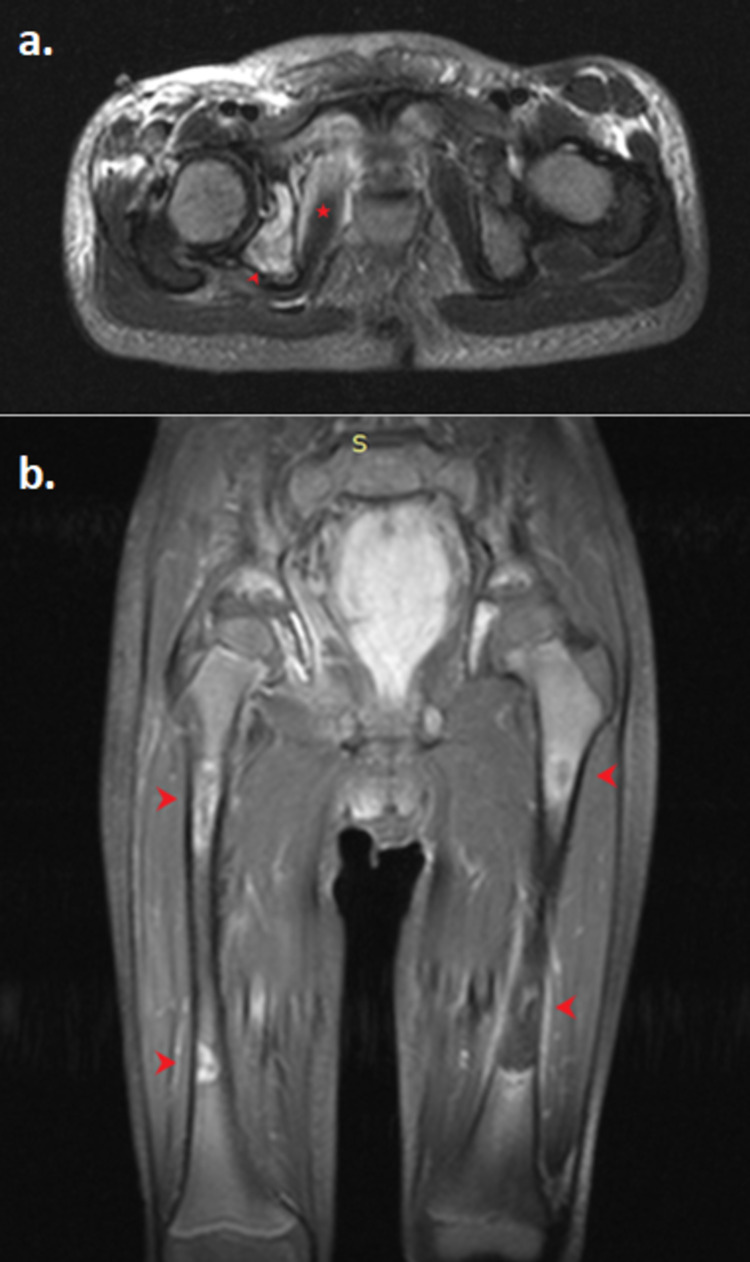
Hip, pelvis, and femur MRI STIR sequence. a) Axial view illustrating increased MR signal and inhomogeneous enhancement of the right acetabulum (arrowhead) and edema of the obturator externus muscle (star) with normal MR signal of the femoral head. b) Coronal view depicting multiple osseous lesions along the femur diaphysis bilaterally (arrowheads). MRI: magnetic resonance imaging, STIR: short tau inversion recovery

**Figure 6:**
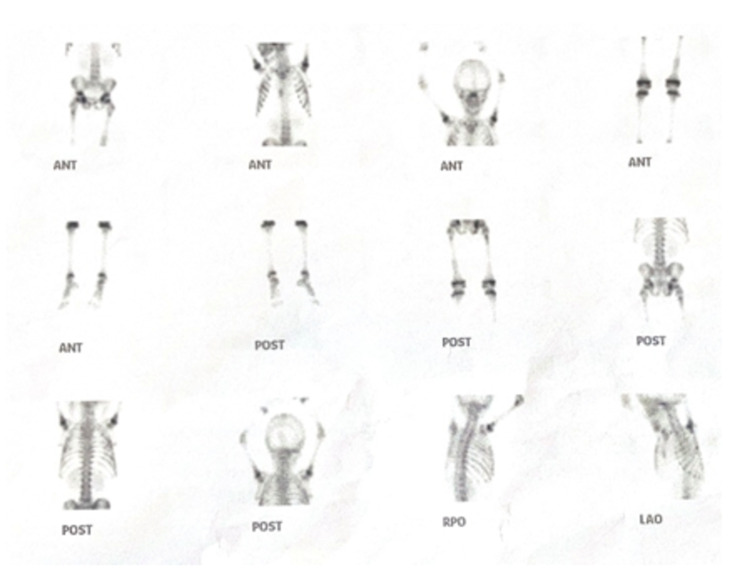
Whole-body bone scan Tc-99m-MDP depicting inhomogeneous tracer distribution at multiple sites. Tc-99m-MDP: technetium-99m methylene diphosphonate, ANT: anterior, POST: posterior, RPO: right posterior oblique, LAO: left anterior oblique

**Figure 7:**
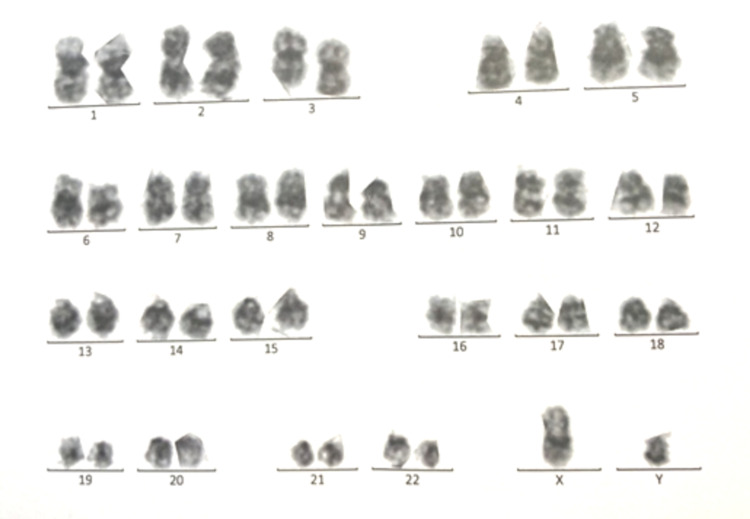
Karyotype – isochromosome 6p.

The patient underwent urgent irrigation and debridement of the right hip joint. Aspiration of synovial fluid sample for culture and synovial biopsy for culture and histological assessment were performed intraoperatively. On days 1 and 2 postoperatively, the boy clinically improved without fever or episodes of right hip pain. Laboratory tests revealed a reduction in WBC count to the normal range and a postoperative CRP level of 156 mg/L. The biochemical profile was normal, except for serum LDH levels, which remained remarkably high, approximately three times higher than normal (Table 1). On day 3 postoperatively, the boy experienced once again subsidence of fever (38.3°C) and RLQ pain. Synovial fluid culture proved to be sterile, and the histopathological evaluation of the synovium biopsy specimen revealed only acute and chronic inflammation. A second-look MRI evinced the presence of increased MR signal and inhomogeneous enhancement of the right acetabulum and obturator internus and externus muscle. In addition, osseous lesions were depicted at both femurs along the diaphysis with a maximum diameter of 4.35 cm (upper third of the left femur diaphysis) (Figure 5). Malignancy was considered as part of the differential diagnosis due to the combination of abnormal MRI findings with LDH elevation, lymphocytosis, and persistent fever. In order to exclude the possibility of malignancy, a triple-phase bone scan with technetium-99m methylene diphosphonate (Tc-99m-MDP) was performed on the fifth postoperative day. Investigation of hip joints demonstrated slightly increased radiotracer uptake, especially on the right side, in the flow and blood pool phase. The osseous phase indicated symmetrical increased uptake in both hip joints with increased osteoblastic activity of the upper third of the right femur. Whole-body skeletal scintigraphy showcased multiple bone lesions with increased or decreased osteoblastic activity across the spine, thoracic cage, sternum, femurs, ilium, ischium, and pubic bones bilaterally (Figure 6). The aforementioned morphology and distribution pattern raised high suspicion for metastatic lesions. Examination of peripheral blood smear highlighted the presence of blasts (18.5%), while flow cytometry immunophenotyping was positive for CD19, CD10, cytCD79a, and terminal deoxynucleotidyl transferase (TdT) and negative for CD45. The case was referred to the pediatric hematology-oncology service of the hospital, which proceeded with a CT-guided core biopsy of the left femur proximal metaphysis. The histopathology evaluation revealed hypercellular bone marrow, reactive marrow alterations with left-shifted granulopoietic maturation, and nonspecific stromal inflammatory reaction. Because the latter was not diagnostic, a bone marrow examination along with immunohistochemistry evaluation was required. The blast percentage was 54.6%, and immunophenotype examination established the diagnosis of a common B-form (B-precursor) ALL. Bone marrow chromosome analysis revealed the presence of isochromosome 6p with a breakpoint at p10 (Figure 7). The reverse transcriptase-polymerase chain reaction (RT-PCR) study did not detect BCR/ABL and MLL/AF4 fusion genes; however, the RT-PCR study for TEL-AML1 fusion gene was positive. Lumbar puncture did not detect blasts in the cerebrospinal fluid. The patient was immediately started on induction chemotherapy, following the ALL IC 2009 protocol, and flow cytometry analysis for bone marrow minimal residual disease (MRD) was negative on day 29. The duration of treatment was two years, and the patient attained complete remission, being disease-free at three years of follow-up.

## Discussion

ALL constitutes the most common pediatric cancer, accounting for approximately 25% of all childhood malignancies, with an incidence of 40 per million cases [2,3]. Regarding pathogenesis, ALL involves the differentiation and proliferation of lymphoid progenitor cells in bone marrow and blood, along with extramedullary organ and tissue infiltration [4]. In the last decade, genomic studies have aided the scientific community to make great progress in decoding and understanding the biological basis of pediatric ALL. Although the majority of ALL cases develop in otherwise healthy humans, specific predisposing factors have been acknowledged. Hereditary susceptibility regarding constitutional syndromes (Down syndrome, Fanconi anemia, Bloom syndrome, ataxia-telangiectasia, and Nijmegen breakdown syndrome) and familial cancer syndromes (Li-Fraumeni syndrome), several gene variants and mutations, polymorphism of single nucleotides, and environmental factors (viruses, pesticides, and ionizing radiation) have been related to leukemogenesis at least in a subgroup of ALL cases [1,5].

The clinical manifestation of ALL can be very heterogeneous between individuals and primarily includes a characteristic constellation of symptoms reflecting bone marrow failure, such as paleness, asthenia, abnormal skin or mucosal bleeding, bruising, petechiae, purpura, anorexia, unintended weight loss, osteoarticular pain, and fever. Organomegaly and lymphadenopathy can be present in up to 68% of patients; however, these extramedullary manifestations due to leukemia cell organ infiltration are usually asymptomatic [6]. Musculoskeletal complaints of ALL may include pain, functional impairment, limping, refusal to bear weight, swelling, and joint effusion, with long bones being more commonly involved than the axial skeleton [7]. The pathophysiology of osseous pain in ALL is attributed to the speedy expansion of bone marrow due to the abnormal proliferation of clonal lymphoid cells within the medullary cavities eliciting a mass effect [8]. Initial presentation with isolated bone or joint pain with normal laboratory features and peripheral blood smear can occur in 15%-30% of ALL cases, while this type of musculoskeletal manifestation is also associated with a low incidence of extramedullary site involvement, making the diagnostic procedure even more complicated [9]. According to a retrospective study of pediatric patients with ALL by Marwaha et al. in 2010, 8.4% presented with musculoskeletal complaints, and 76.5% of them were at first misdiagnosed with juvenile idiopathic arthritis (JIA), with asymmetrical oligoarticular arthritis of the large joints being the most common pattern [10].

Regarding the laboratory data, CBC routine test may reveal cytopenia; however, a retrospective study by Jonsson et al. found that patients with ALL with predominant bone pain presented nearly normal hematological indexes, resulting in a delayed diagnosis [11]. In addition, Hara et al. in their retrospective study reported that ALL pediatric patients who presented with a PLT count of >150 × 10^3^ (17.8%) tended to also present less remarkable anemia (p < 0.05) and leukocytosis (p < 0.01) [12]. Inflammatory markers are typically elevated, but these parameters can be misleading as rheumatic and infectious diseases also present with elevated CRP and ESR values. Another indicator of utmost importance to distinguish a malignant neoplasm among other conditions, especially rheumatologic disorders, that manifests with joint pain is the serum LDH value; 2.2 times higher LDH value with a normal hematological profile is highly suggestive of malignancy, while 0.8 times elevated LDH level preferably indicates a rheumatic disorder, although a normal LDH value cannot preclude malignancy [13]. Usually, the microscopy of a peripheral blood smear will indicate the presence of blast cells confirming the diagnosis; however, the absence of blasts cannot rule out leukemia.

In the event of ALL presenting with osseous pain, radiological assessment can be useful as it may reveal periosteal reaction with new bone formation, osteolytic/sclerotic or mixed lesions, radiolucent metaphyseal bands, permeative bone destruction, osteopenia, and pathologic fractures; however, none of the aforementioned radiographic abnormalities are pathognomonic for ALL [2,14]. According to a retrospective review of 122 children with ALL by Sinigaglia et al., 38.3% of patients had musculoskeletal symptoms at presentation, and 40.2% of this subgroup had at least one abnormal X-ray finding at this time, with the most common being the osteolytic pattern [15].

Childhood ALL treatment involves three phases: induction, consolidation, and maintenance therapy, with a maximum duration of two and a half years. Along with the conventional approach of chemotherapeutic agents, new strategies such as immunotherapy and molecular targeted therapy have come up, with hematopoietic cell transplantation (HCT) being utilized for very high-risk patients. Children with ALL are divided into three risk groups (low, high, and very high risk) based on certain prognostic factors in order to be enrolled in risk-based treatment protocols. Age, WBC count at diagnosis, race, sex, T-cell or B-cell immunophenotype, central nervous system involvement, MRD assessment, specific genetic alterations-translocations, hyperdiploidy/hypodiploidy, and response to initial chemotherapy are all factors taken under consideration for treatment assignment [6]. Prior to the introduction of chemotherapy, the five-year survival rate of children with ALL was 4% [2]. Currently, the improvement of previous and development of novel treatment stratification has remarkably increased the five-year survival rate to 90%, with lower-risk patients having a better prognosis [16].

In the setting of an acutely limping child, vigilance is warranted by the clinician. First of all, the age of the patient can help in the differential diagnosis, as certain conditions such as slipped capital femoral epiphysis and Legg-Calve-Perthes disease present more often in adolescents. Migratory joint pain, morning pain, and stiffness should make the caretaker think of a rheumatoid process, while night pain interrupting sleep and constitutional symptoms should raise suspicion of an underlying malignancy. In addition, referred pain from the spine or knee must be taken under consideration during a meticulous physical examination. Differentiation between transient synovitis and septic hip can be challenging, as the range of motion can be restricted in both conditions. If infection is suspected, the assessment of CBC, CRP, and ESR and joint aspiration can help in establishing a definite diagnosis. Although ultrasound can detect the presence of a minor effusion, it cannot distinguish septic from reactive effusion. Plain radiographs of the area of interest should always be ordered as they could exclude or verify the presence of a fracture or reveal joint space narrowing, hip dysplasia, slippage of the femoral head, or bone fragmentation.

In this article, we highlight the case of a child with refractory hip pain and fever with initially non-obvious hematological aberrations except for remarkable elevated inflammatory markers that were managed as septic arthritis. Septic arthritis constitutes an orthopedic emergency that necessitates prompt intervention, as delayed management can lead to joint degeneration, loss of motion, and functional impairment. Even sterile cultures from joint aspiration cannot rule out septic arthritis, considering that approximately 50% of cases of bacterial arthritis are proven negative [17]. An upcoming short clinical course of improvement after irrigation and debridement of the patient’s hip joint along with IV antibiotics misled us to assume that our diagnosis was accurate. The subsequent relapse of symptoms, concomitantly with lymphocytosis, anemia, significant serum LDH elevation, and inguinal lymphadenopathy that was not palpable, raised the clinical suspicion of malignancy. Eventually, the bone marrow biopsy galvanized the establishment of a definite diagnosis of ALL.

## Conclusions

In the acute presentation of a child with musculoskeletal symptoms, red flags indicative of a possible diagnosis of leukemia are apparent deviations of the three major cell lineages and significant serum LDH elevation along with symptoms reflecting hematopoietic insufficiency and leukemic organ infiltration. Of note, acute leukemia with dominant orthopedic complaints will less commonly present with abnormal hematological indexes and organ infiltration, so clinical alertness is required for prompt diagnosis and management. A bone marrow biopsy or the identification of blasts in peripheral blood smear will eventually confirm the diagnosis.
